# Dysregulation profile in children of ethnoracially diverse at-risk families: Factor structure and longitudinal correlates

**DOI:** 10.1017/S095457942300007X

**Published:** 2023-02-27

**Authors:** Hali Kil, Charlotte Longpré, Geneviève A. Mageau

**Affiliations:** 1 Department of Psychology, Simon Fraser University, Burnaby, Canada; 2 Department of Psychology, Université de Montréal, Montréal, Canada

**Keywords:** at-risk youth, dysregulation, fragile families, parenting, strengths

## Abstract

The present work sought to confirm the factor structure and examine longitudinal strengths-based and mental health correlates of the dysregulation profile (DP) in children of at-risk fragile families of diverse ethnoracial backgrounds. The data came from the Fragile Families and Child Wellbeing Study (*N* = 2125 families). Mothers (*M*_age_ = 25.3) were mostly unmarried (74.6%), and children (51.4% boys) were identified as Black (47.0%), Hispanic (21.4%), White (16.7%), or multiracial or other backgrounds. Childhood DP was constructed using mother reports of the Child Behavior Checklist at age 9. Mothers’ in-home parent–child interactions and depressive symptoms were assessed at child age 5. At age 15, children responded about their own mental health, social skills, and other strengths-focused outcomes. A bifactor DP structure fit well to the data, with the DP factor representing difficulties in self-regulation. Using SEM, we found that mothers who were more depressed and used less warm parenting at child age 5 had children who presented with higher DP at age 9. DP was in turn associated with less social skills, perseverance, optimism, and more anxiety, depression, and impulsivity at adolescence. Childhood DP appears to be relevant and applicable for at-risk, diverse families, and may also impede on children’s future positive functioning.

The Dysregulation Profile (DP) is an index of overall disrupted self-regulation that captures regulation problems common to general emotional, attentional, and behavioral difficulties in children and youth (Deutz et al., 2018; Geeraerts et al., 2015). The DP has received increasing attention among clinical and applied psychologists as the index serves as an important precursor for later development of psychopathology, including increased risks of mood and behavior disorders (Althoff et al., 2010; Bellani et al., 2012; Holtmann et al., 2011). Emerging work has also turned to parenting antecedents such as harsh parenting that may play a direct role in the development or maintenance of elevated DP in childhood (e.g., Aebi et al., 2020; Kim et al., 2012).

However, the DP and its potential antecedents or outcomes have not yet been examined in underrepresented groups, such as at-risk ‘fragile’ families, typically representing low income, single parent families with low parental educational attainment. These families largely consist of ethnoracial minorities, such as families with Black and Hispanic backgrounds in the USA, who are typically underrepresented in clinical research. A directed assessment of the DP and its potential antecedents and outcomes in these families may help to identify the relevance of the DP for children of these families and highlight protective factors that can alleviate the developmental risks leading up to reduced social and emotional adjustment in later years. Further, such work would also help to answer recent calls for more diverse perspectives in clinical settings (Moreno & Chhatwal, 2020), including low SES background families. Thus, in the present study, our aims were to examine the DP in this group of diverse fragile families, as well as to examine potential parental antecedents and adolescent outcomes, particularly strengths-based outcomes, associated with heightened DP at childhood.

### The dysregulation profile

Knowledge on child mental health difficulties and psychopathology has blossomed over the past few decades (McLeod, Weisz, et al., 2007; McLeod, Wood, et al., 2007; Rothbaum & Weisz, 1994). The focus in past research has been largely limited, however, to specific internalizing and externalizing child outcomes. An emerging area of work has focused on the DP as a marker of generalized regulatory problems in childhood and youth, marked by high scores on the anxious/depressed, attention problems, and aggressive behaviors scales (i.e., AAA scales; Achenbach & Rescorla, 2001). As a regulation difficulty that manifests beyond the specific AAA scales (Deutz et al., 2016), DP identifies a phenotype of poor regulation of emotions, attention, and behavior in children (Ayer et al., 2009). Given its links to psychiatric disorders and addiction later in life, DP is considered a developmental index for major psychopathology in emerging adulthood (Bellani et al., 2012).

Studies validating the factor structure of the DP based on AAA scales in both clinical and community samples have examined various structural models, with all converging on a bifactor model as best fitting (Althoff et al., 2010; Deutz et al., 2016; Geeraerts et al., 2015; Holtmann et al., 2011). In the bifactor model, DP is designated as a general factor (factor 1) with three other specific factors for the AAA scales, respectively (factor 2). The bifactor structure has been validated across various informants (mother, father, teacher and youth reports; Althoff et al., 2010, Deutz et al., 2016), and across different questionnaires employed to assess the DP, including the Child Behavior Checklist (Deutz et al., 2018; Holtmann et al., 2011). The bifactor model illustrates the etiology of DP as a comorbidity that cross-cuts characteristics of the AAA scales (Geeraerts et al., 2015) and is thus considered an index of psychopathology (lack of regulation) instead of addressing its specific components, i.e., the AAA scales (Bellani et al., 2012).

We note here that discussions surrounding the value of the bifactor model of DP remain nuanced. In particular, the bifactor model almost always fits the data better than a one-factor model, which includes just the DP factor without the subscales, or a second-order model in which the AAA subscale factors load onto a second-order DP factor (Bornovalova et al., 2020; Watts et al., 2019). Thus, even in cases in which the bifactor model fits the data best, the model may not follow a bifactor structure. Accordingly, recent works have called on researchers to calculate reliability of the latent factors in bifactor models to assess the value of separately assessing the subscale factors and general factors, in this case, AAA subscales and DP, respectively (e.g., Rodriguez et al., 2016).

The premise that follows from the DP bifactor model is that separating the DP and the AAA residuals factors can provide information on the underlying developmental difficulties experienced across different mental health symptom groups, as captured by the general DP component independent of what is unique to these symptom groups (Bornovalova et al., 2020). Accordingly, Deutz et al. (2016) suggest that a bifactor structure highlights DP as a general syndrome that cross-cuts specific groups of symptomatology, disentangling the shared and unique variances of the DP factor from the AAA scales while modeling the shared variance between items from the same subscale. As such, DP may represent a crosssymptomatic index of difficulties with self-regulation (i.e., dysregulation), while the AAA residuals may represent the corresponding arousal that may be present for each specific symptom group. Thus, the bifactor model can be valuable for delineating the general, underlying difficulties in regulation (DP) from the specific issues that mark the different domains in which dysregulation may manifest, such as attention difficulties or internalizing and externalizing problems.

### At-risk ragile families

To date, the samples of focus in studies examining the DP have been largely limited to European descent and White participants (approx. 60%–90% of sample) and middle to upper-middle class families (Deutz et al., 2016, 2020; Geeraerts et al., 2015; Keefer et al., 2020; Kim et al., 2012; McQuillan et al., 2018). Further, much of this work has been focused on clinically at-risk children who have received diagnoses or clinic referrals for mental health difficulties, such as mood, attention, and behavioral disorders (Halperin et al., 2011; Haltigan et al., 2018; Mbekou et al., 2014). For example, Jucksch et al. (2011) found in a large sample of children receiving services at psychiatric hospitals that childhood DP (as measured by T-score cutoffs) was associated with elevated psychosocial difficulties and maladjustment, more so compared to children with attention problems, attention and mood problems, and clinical controls.

Although a recent multi-site latent class analysis of the CBCL showed prevalence of the DP in 29 different societies (Rescorla et al., 2021), without knowledge of differential antecedents and outcomes, the applicability of the DP for various socioeconomic groups, particularly in multicultural societies with economic class divides, is not clear. Understanding whether the DP is applicable to socioeconomically at-risk families is particularly important considering that multiple risk factors can compound to increase family dysfunction and poor psychosocial outcomes in children from these families (Wade et al., 2014). Indeed, according to the Family Stress Model (Masarik & Conger, 2017), families facing economic hardship and related psychological stress exhibit more disrupted parenting, culminating in child adjustment problems.

At-risk fragile families represent one group of such families that may experience heightened family distress. These families include mothers who are at higher risk of single motherhood, with low income and education, and consist largely of ethnoracial minority families, such as families with Black and Hispanic backgrounds in the USA (Reichman et al., 2001). In addition to financial instability, parents in these families also experience heightened parenting-related stress due to juggling numerous responsibilities relating to childcare, with stress sometimes spilling over into maladaptive and harsh parenting behaviors (Shelleby, 2018). Further supporting the Family Stress Model, child regulation issues, as well as other psychopathological symptoms, are on average more frequently reported in children from fragile families (McLanahan et al., 2019; McLanahan & Beck, 2010). Given these elevated developmental risks in children of fragile families, examining the relevance of DP in these children may provide valuable information on their developmental needs. As the factor structure of the DP has yet to be examined in a diverse group of at-risk families, the present study sought to confirm that the bifactor structure would fit best in this sample, following the steps taken by Deutz et al. (2016) and others (e.g., Geeraerts et al., 2015).

### Antecedents and outcomes of the dysregulation profile

Beyond factor structure, research has also expanded upon the potential antecedents and outcomes of the DP in childhood. Regarding antecedents, research suggests that there may exist genetic and environmental explanations for the development of dysregulation more generally. While genetic accounts focus on the heritable and biological nature of regulation difficulties, environmental accounts focus on the familial and contextual factors that may contribute to dysregulation as children develop. For example, Beauchaine (2015) explains in his biosocial developmental model of emotion dysregulation that for children with inherited genetic risk for impulsivity, parenting that is highly coercive or controlling can impede the development of self-regulation skills, ultimately resulting in children’s chronic dysregulation.

In the present work, we build on existing literature on the parental characteristics that may facilitate or protect against dysregulation. For example, Kim (2012) showed that DP in 549 pre-schoolers from the general population in the USA was linked to maladaptive and harsh parenting. Moreover, a longitudinal study conducted by Deutz et al. (2020) reported a number of parental factors predictive of DP in 1073 children in the USA, including low family income, low maternal education, less positive maternal parenting, and poorer home environment. Further, maternal depression has been found to be a significant longitudinal predictor of heightened DP in multiple studies (e.g., Deutz et al., 2020; McQuillan et al., 2018). These parental risk factors of DP have been found to be elevated in at-risk fragile families. For example, mothers from fragile families have been shown to more often use harsh and aggressive parenting, less positive parenting, and face greater risk for depression (Lanier et al., 2014; Reichman et al., 2001; Shelleby, 2018). Genetic and environmental factors may also cumulate to increase the risk of DP in at-risk children: mothers with regulatory difficulties have been shown to use more negative parenting (environmental) and their children may also inherit their mothers’ genetic characteristics that mark dysregulation (see Beauchaine, 2015). Overall, a confirmatory examination of how these parental characteristics may contribute to the development of DP in children from fragile families is warranted.

Various psychopathological outcomes have also been associated with DP in adolescence and early adulthood. Childhood DP has been connected to later indicators of comorbid mood disorders such as anxiety and depression, attention deficit hyperactivity disorder, as well as suicidal thoughts and behaviors in adolescence (Althoff et al., 2010; Deutz et al., 2016; Meyer et al., 2009). Other studies have identified adolescent outcomes such as loneliness, poor psychosocial maturity, indicators of aggression, and various personality pathologies in relation to childhood DP (Deutz et al., 2018, 2020). Collectively, these findings suggest that DP in children may be associated with heightened mental health problems in adolescence.

However, less is known about how childhood DP can limit the development of positive psychosocial outcomes. Only one recent work has examined a positive social outcome in adolescence – namely, friendship quality – which was negatively linked to childhood DP (Deutz et al., 2020). In line with perspectives of strengths-focused researchers such as Zimmerman (2013), understanding strengths-based child outcomes in at-risk populations can elicit important insights into how to promote children’s positive development, beyond preventing negative outcomes such as mental health difficulties. Given that children from fragile families may face heightened developmental difficulties in psychosocial adjustment and mental health (Yoshikawa et al., 2012), these insights may be valuable to illuminate the pathways through which preventive interventions for parents may help to promote positive adolescent outcomes in this population.

### The present study

The aims of this study were thus twofold. First, we intended to confirm the factor structure of the DP in a sample of children from fragile families. Given that most existing work on the DP has focused on mostly White, European-descent children from middle or higher socioeconomic status families, the present work would verify that the often-used bifactor model identifying DP as unique from the AAA subscales would be relevant particularly for ethnoracial minority and socioeconomically at-risk children. We followed the guidelines for examining factor structure of the DP as described by Deutz et al. (2016) and others (e.g., Geeraerts et al., 2015). Echoing these works, we hypothesized that the bifactor structure would fit best, representing common variance related to self-regulation (DP) across the AAA subscales.

Second, we examined maternal antecedents (depression, observed parenting) at child age 5 and adolescent outcomes at child age 15 that may correspond with DP in this sample. We focused on parenting at age 5 due to the overlap with age 9 with regards to children’s increasing interactions with peers and other socialization figures (preschool and elementary school, respectively). We expected that the strategies parents use at age 5 when children begin to interact with others beyond the home would be associated with their regulation by age 9. Additionally, we followed perspectives by researchers that socialization is an ongoing process throughout childhood (e.g., Zhang et al., 2020; meta-analysis by Karreman et al., 2006). In particular, we aimed to explore strengths-based adolescent outcomes such as adaptive social skills, task engagement, perseverance, optimism, connection to others, and happiness, in addition to adolescent difficulties such as anxiety and depression symptoms and impulsivity. We hypothesized that more maternal depression, more negative and less positive observed parenting would be related to higher dysregulation. We further expected that higher dysregulation may be related to reduced psychosocial strengths and higher anxiety, depression, and impulsivity at adolescence.

## Method

### Participants and procedures

Participants were mothers and children from the Fragile Families and Child Wellbeing Study dataset (FFCWS; Reichman et al., 2001). The FFCWS is a longitudinal project that includes families of children across 20 US cities, and oversampled unmarried m others. Between 1998 and 2000, 4989 biological mothers were recruited and interviewed across 75 hospitals at the birth of the focal child. Follow up interviews were conducted at child ages 1, 3, 5, 9, and 15. In-home assessments were also conducted in the latter four waves of data collection. In the present study, the selected parenting measure was taken from the in-home assessments, for which the research team selected only one primary caregiver for coding of parenting behaviors. In most cases (over 90%), mothers were the primary caregiver. Thus, we selected only those families in which mothers participated in in-home assessments at the relevant waves of data collection (child ages 5, 9, and 15), so as to maximize sample size while accounting for maternal continued involvement in the child’s life across the three timepoints.

Using the above criteria, 2,191 mothers and their children were identified. Of this group, 2,125 mothers had valid data, thus comprising the final selected sample. All mothers in this final sample provided consent to participate. At the birth of the child, mothers were 25.33 years old on average (SD = 6.06). Less than half (38.9%) had at least some college education, and average household income was $32,950.68 (SD = $31568.39). As a reference, in 2,000, 51.8% of the US adult population (age 25+) had at least some college education and the median household income was $42,148 (U.S. Census Bureau, 2001, 2003). Most mothers (74.6%) were not married to the child’s father, and only 40.1% of mothers were neither married nor living with the father. Mothers’ ethnoracial backgrounds were mostly Black (51.8%), Hispanic (22.7%), and White (22.2%), with a smaller proportion of ‘other’ backgrounds (3.2%). Child gender was approximately equal, with 51.3% boys. Children reported their own ethnoracial background at age 15, with most children reporting Black (47.0%) and Hispanic (21.4%) backgrounds, fewer as White (16.7%), and smaller proportions of multiracial (4.6%) and ‘other’ (2.5%) backgrounds.

The study protocol and procedures were approved by the Princeton University Institutional Review Board. All participants gave written and informed consent to participate, and received compensation ($100 for parents, $50 for adolescents).

### Measures

#### Child behavior checklist (*age 9*)

The subscales for Anxious/Depressed (13 items; e.g., worries, cries a lot), Attention (10 items; e.g., can’t concentrate, impulsive or acts without thinking), and Aggression problems (18 items; e.g., destroys things, gets in fights) from the parent-reported Child Behavior Checklist (CBCL; Achenbach & Rescorla, 2001) 6–18 were used. Items were rated on a 3-point scale from 0 = not true to 2 = very true or often true. Conceptualization of the DP is further described in the Analytic Plan.

Interitem reliabilities as demonstrated by Cronbach’s alpha (*α*) and intercorrelations for all continuous measures are depicted in Table 1. We note that Cronbach’s alpha scores tend to be low in scales consisting of fewer than 10 items (Pallant, 2007; Sijtsma, 2009), and thus included scale variables with scores lower than .7 in proceeding with analyses.

#### Parental antecedents (*age 5*)

##### Maternal depression.

Questions pertaining to major depressive episodes for mothers were asked using the Composite International Diagnostic Interview–Short Form (World Health Organization, 1990). Validity, test-retest reliability, inter-rater reliability of the measure have been established in existing research (Kessler et al., 1998; Wittchen, 1994). Consistent with diagnostics in the DSM 4^th^ Edition, mothers were first asked if they had feelings of dysphoria or anhedonia in the past year lasting for at least two weeks. If mothers endorsed these feelings, they were asked whether seven symptoms were present during that time (e.g., trouble with sleep; feeling down), 1 = Yes, 2 = No. Endorsing at least three symptoms as Yes (1) was categorized as meeting depression criteria, with each participant coded as 0 = does not meet criteria, 1 = meets criteria. In this sample, 16.4% of mothers met criteria for depression.

##### Parenting.

Observed parenting behavior was assessed using an adapted version of the Home Observation for Measurement of the Environment (HOME; Bradley, 1993). The original HOME has been validated in diverse family settings (see review by Totsika & Sylva, 2004). The observational measure was taken by research team members during the in-home interview. Two averaged subscales with dichotomous scoring (0 = not present, 1 = present) were used in the present study: the 8-item Parental Warmth subscale (e.g., parent voices positive feelings to child; parent caresses, kisses, or hugs child), and the 3-item Parental Hostility subscale (e.g., parent expresses overt annoyance with child; parent slaps or spanks child).

#### Adolescent outcomes (age 15)

##### Adaptive social skills.

Items from two scales adapted for adolescent self-report composed the measure of adaptive social skills. Three items were taken from the Adaptive Social Behavior Inventory (ASBI; Hogan et al., 1992), and assessed adolescents’ understanding of others’ feelings, sympathetic behavior, and openness and directness. Nine items were taken from the Assertion scale of the Social Skills Rating System (SSRS, now called the Social Skills Improvement System-Rating Scales; Gresham & Elliott, 1990, 2008), and assessed adolescents’ social self-confidence, adaptability, and initiative (e.g., I make friends easily; I am liked by others). Validity and reliability for the ASBI (Dearing et al., 2001; Eşkisu & Kapçı, 2021; NICHD Early Child Care Research Network, 1998) and the Assertion subscale of the SSRS (P. P. P. Cheung et al., 2017; Gresham et al., 2010, 2011) have been established in previous literature, although ASBI has been limited to samples with younger children in existing work. Items were rated on a 3-point scale from 1 = not true to 3 = often true, and all 11 items were averaged for an overall social skills score, as suggested by the FFCWS research data guide.

##### Positive functioning.

The 20-item EPOCH Measure of Adolescent Wellbeing (Kern et al., 2016) was used to assess self-reported adolescent positive functioning. Based on the PERMA model of adolescence proposed by Seligman (2012), the EPOCH consists of five 4-item subscales: engagement (e.g., I get completely absorbed in what I am doing; I get so involved in activities that I forget about everything else), perseverance (e.g., I finish what I begin; once I make a plan to get something done, I stick to it), optimism (e.g., in uncertain times, I expect the best; I think good things are going to happen to me), connectedness (e.g., when I have a problem, I have someone who will be there for me; I have friends that I really care about), and happiness (e.g., I feel happy; I am a cheerful person). Previous work has established good to excellent interitem reliability, test-retest reliability, factor structure, and validity (Kern et al., 2016; Taheri et al., 2021; Zeng & Kern, 2019). Items on each subscale were rated on a 4-point scale from 1 = strongly agree to 4 = strongly disagree, then reverse coded and averaged such that higher scores indicated more positive functioning.

##### Anxiety.

The 6-item anxiety subscale of the Brief Symptom Inventory (BSI; Derogatis & Melisaratos, 1983) was used to assess adolescents’ self-reported anxiety symptoms in the last four weeks (e.g., have spells of terror or panic; feel nervous or shaky inside). The BSI is well-validated and shows excellent reliability, including in adolescent samples (Cooper et al., 2003; Derogatis & Savitz, 2000; Piersma et al., 1994). Items were rated on a 4-point scale from 1 = strongly agree to 4 = strongly disagree, then reverse coded and averaged such that higher scores indicated more anxiety.

##### Depression.

Five items from the Center for Epidemiologic Studies Depression Scale (CESD; Radloff, 1977) were used to measure adolescent depressive symptoms in the past week (e.g., feel sad; feel life is not worth living). This 5-item version of the CESD was formerly used in the National Longitudinal Study of Adolescent Health and is cross-culturally validated (Perreira et al., 2005). Items were rated on a 4-point scale from 1 = strongly agree to 4 = strongly disagree, then reverse coded and averaged such that higher scores indicated more depression.

##### Impulsivity.

Six items taken from Dickman’s Impulsivity Scale (Dickman, 1990) were used to assess adolescent impulsivity (e.g., Often, I don’t spend enough time thinking over a situation before I act; I often get into trouble because I don’t think before I act). Validity and reliability have been established in existing work, including for adolescents (Fino et al., 2014; Pechorro et al., 2021). Items were rated on a 4-point scale from 1 = strongly agree to 4 = strongly disagree, then reverse coded and averaged such that higher scores indicated more impulsivity, as suggested by the FFCWS research data guide.

#### Analytic plan

**Figure 1.**
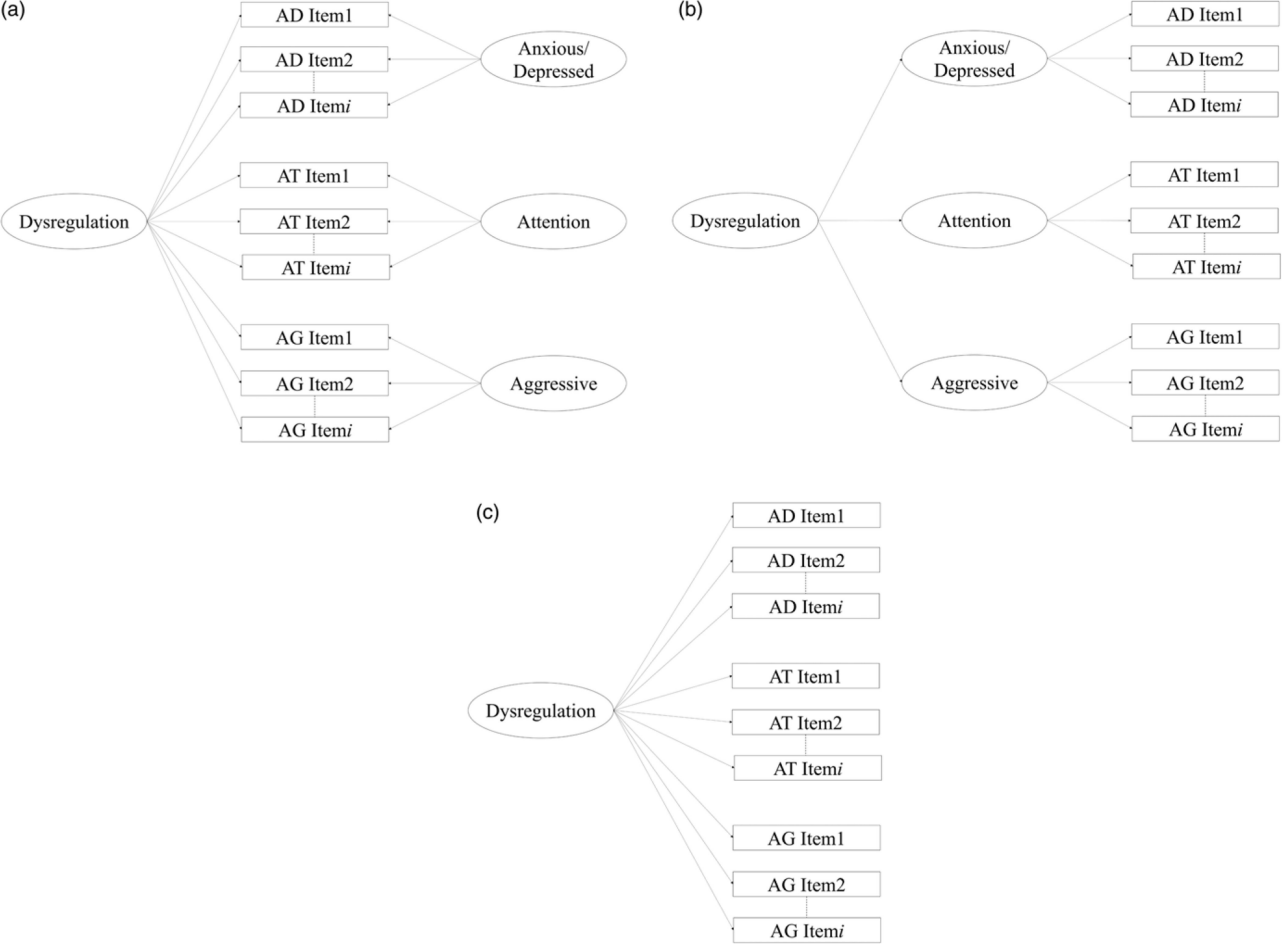
Three potential factor structures of the Dysregulation Profile: *a* represents the second order model, *b* represents the one factor model, and *c* represents the bifactor model. AD represents Anxiety/Depression, AT represents Attention Problems, and AG represents Aggression.

First, we verified the factor structure of the DP following steps taken in earlier studies. Three competing structures were tested in *Mplus 7*: (a) the bifactor model, in which each AAA subscale set of items loaded onto its corresponding latent subscale factor in addition to all items loading onto the latent factor DP, for a total of four uncorrelated latent factors at the first order level; (b) the second order model, in which each AAA subscale set of items loaded onto its corresponding latent subscale factor, which in turn loaded onto the higher order latent factor DP, for a total of three first-order and one second-order factors; (c) the one factor model, where all AAA items loaded onto a single latent first-order factor of DP. The three structures are depicted in Figure 1.

CBCL items were dichotomized (0 vs. 1 and 2), consistent with previous work examining the factor structure of the CBCL-DP (Deutz et al., 2016), as our community-based sample reflected similar issues of zero-inflation in descriptive analyses (up to 90% for some items). Using the weighted least square mean and variance adjusted (WLSMV) estimator, pairwise deletion was automatically implemented for missing data; however, covariance coverage was very high (at least 98%), suggesting few missing values. We determined fit based on the following model fit criteria: Root Mean Square Error of Approximation (RMSEA) < .08, Comparative Fit Index > .95, and Weighted Root Mean Square Residual close to 1.00 (Hooper et al., 2008). Although we report the *χ*^2^ test, with smaller *χ*^2^ values suggesting better fit, we did not use the *χ*^2^ test as a model fit criterion due to the large sample size represented in our dataset. Model comparisons were based on CFI and RMSEA, with ΔCFI = .01 and ΔRMSEA = .015 indicative of significant difference between models (Byrne & Stewart, 2006; G. W. Cheung & Rensvold, 2002). We further considered confidence intervals (CIs) of RMSEA, for which significance would be evident by the absence of overlap in CIs between two models. Reliability of latent factors in SEM was also reported using omega (*ω*) and the H coefficient. Omega values of .75 or higher (Hammer & Toland, 2017; Rodriguez et al., 2016), and *H* coefficient values of .70 or higher indicate acceptable reliability (Hancock & Mueller, 2001; Rodriguez et al., 2016).

Second, we examined the parenting antecedents and adolescent psychosocial outcomes related to the selected DP structure. All variables were entered into a single structural equation model (SEM) in *Mplus* using the WLSMV estimator to test the proposed relations simultaneously. Direct paths were added from maternal antecedents (age 5) on each factor of the DP structure (age 9); from the DP structure (age 9) on adolescent outcomes (age 15); and from the maternal antecedents (age 5) on adolescent outcomes (age 15) to account for indirect effects in the relations between maternal antecedents and adolescent outcomes. Several covariates were entered into the model: AAA subscale mean scores at child age 5 (each consisting of a select number of CBCL items), mothers’ age at child’s birth, mother-reported household income, child sex, and child age (in months) at age 9 and 15. The same model fit criteria used above for factor structure validation were applied to assess model fit of the SEM.

## Results

### Descriptive statistics

Table 1 presents descriptive statistics and correlations for all variables of interest. Means indicated that the sample presented high levels of parental warmth and low levels of parental hostility, low levels of child difficulties on the CBCL, moderate to high levels of adolescent positive psychosocial outcomes, and low levels of adolescent mental health difficulties. Correlations were largely significant in the expected directions, with more warm and less hostile parenting and less maternal depression correlated with fewer child difficulties on the CBCL as well as better social skills and less impulsivity at adolescence. Childhood difficulties on the CBCL were significantly correlated with lower levels of psychosocial strengths and higher levels of anxiety, depression, and impulsivity at adolescence.

### Factor structure of DP

Model fit statistics for the three factor structures are presented in Table 2. The data fit best as a bifactor structure, echoing previous work (e.g., Deutz et al., 2016) and confirmed by ΔCFI and ΔRMSEA compared to the two subsequently tested models. As indicated in Table 2, reliability indices of *ω* and the *H* coefficient were satisfactory for all factors, except for the *H* coefficient of the aggression subscale factor, which did not meet cutoff but was close to satisfactory (*H* = .69). Factor loadings for the DP factor were also well-balanced in representation from all items in all AAA subscales, as depicted in Supplemental Table 1. The bifactor structure suggests that all items of the AAA subscales share common variance, interpreted as DP, and that each AAA subscale’s items also share unique variance (AAA scale residuals) independent from DP. Thus, for the present sample of children from fragile families, a common factor can be extracted from the AAA subscale items, suggesting that DP may represent a valid marker of dysregulation.

### Antecedents and outcomes

**Figure 2.**
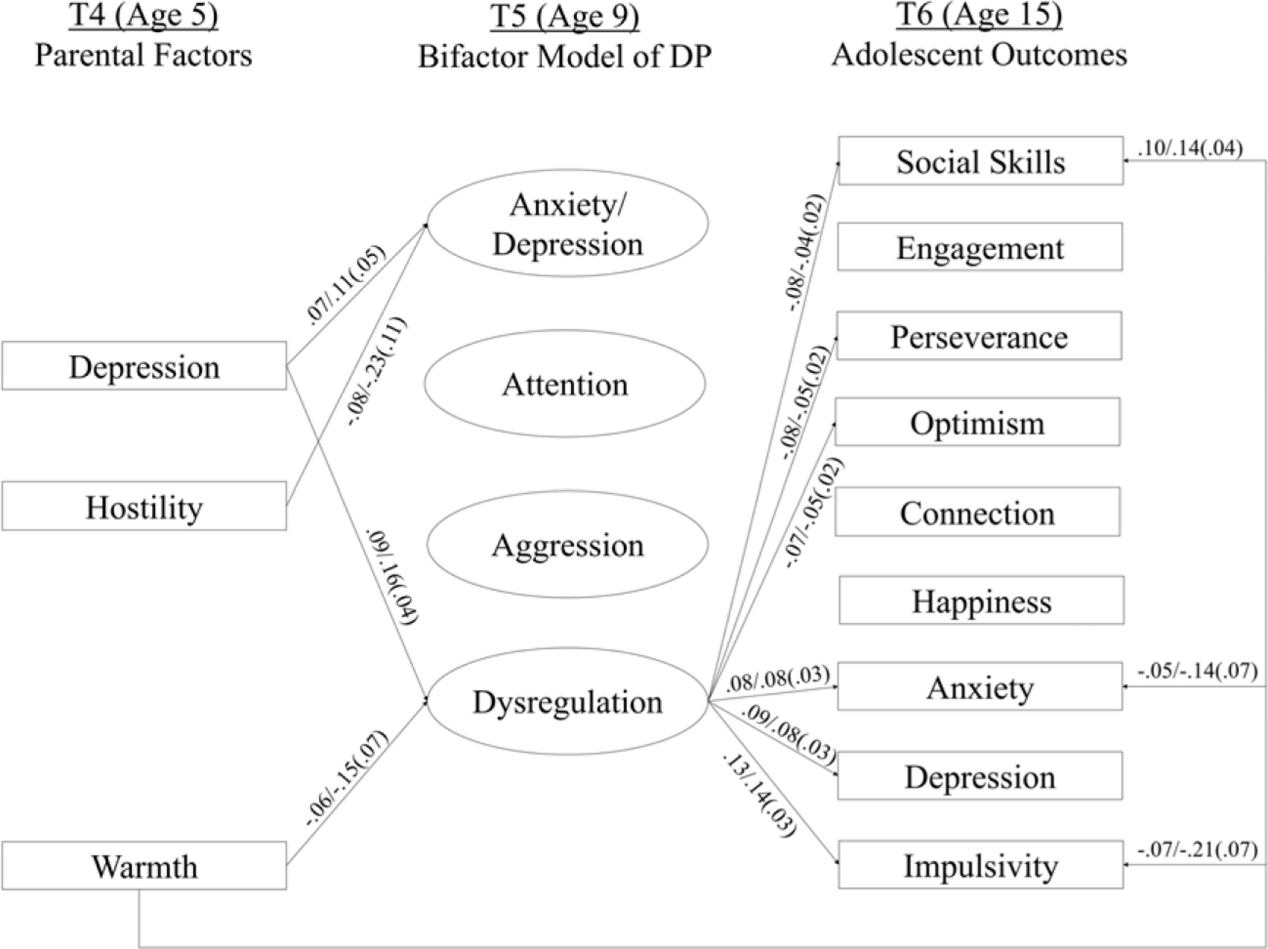
Tested SEM model depicting parental factor antecedents and adolescent outcomes of DP at child age 9. Standardized paths are depicted before the slash and unstandardized paths with standard error in brackets are presented after the slash.Only significant paths at *p* < .05 are presented.

The tested model showed acceptable fit to the data, *χ*^2^(1515) = 3194.62, *p* < .001, RMSEA = .023 (90% C.I. = .022, .024), CFI = .95, WRMR = 1.32. All path estimates are depicted in Supplemental Table 2. As depicted in Figure 2, parents who were more depressed and who used less warm parenting at child age 5 reported more dysregulation at age 9. Dysregulation at age 9 was significantly associated with a number of adolescent outcomes at age 15. Higher dysregulation was linked to less adaptive social skills, less perseverance, lower optimism, heightened anxiety and depression symptoms, and more impulsivity. No other adolescent outcomes were significantly related to dysregulation at age 9, *ps* ≥ .09.

Additionally, parents who were more depressed and used less hostile parenting at child age 5 had children with smaller residuals on anxiety/depression problems at age 9. No other associations between parental factors at child age 5 and child difficulties residuals at age 9 were significant, *ps* ≥ .06. Children’s anxiety/depression, attention, or aggression problems residuals at child age 9 were also not significantly associated with any of the adolescent outcomes, *ps* ≥ .06.

Select direct paths from parental factors at child age 5 to age 15 adolescent outcomes were also significant. More warm parenting was associated with adolescents’ better social skills, *B* = .14, *SE* = .04, p < .001, lower anxiety, *B* =−.14, *SE* = .07, *p* = .04, and less impulsivity, *B* =−.21, *SE* = .07, *p* = 005. Given that DP was also associated with these three outcome variables, indirect paths from warmth on the three adolescent outcomes via age 9 DP were explored. However, none of these indirect paths were significant, *ps* ≥ .06. No other direct effects from parental factors at child age 5 to adolescent outcomes at age 15 were significant, *ps* ≥ .09.

## Discussion

The present study sought to examine the DP, its potential parental antecedents, and adolescent outcomes in a large sample of children from at-risk fragile families, consisting of families of ethnoracially diverse backgrounds and with greater likelihood of single motherhood and low socioeconomic status. Our first aim was to assess the factor structure of the DP in this sample, following established standards for verifying structure (Deutz et al., 2016) and analyzing reliability of the factors in the tested structure (Rodriguez et al., 2016). As hypothesized, we found that a bifactor model fit the data better than a second order or single factor model. Echoing previous research with clinical samples and largely White and Europeandescent samples (e.g., Deutz et al., 2020), the present findings suggest that the DP captures a distinctive common feature of emotional, attentional, and behavioral regulation problems. This finding was bolstered by the reliability scores for the DP general factor, which were strong and, for the *H* coefficient, higher for the DP than for the AAA subscale factors, suggesting that the DP reflects general dysregulation that cross-cuts the specific groups of symptoms represented by the AAA subscales (as suggested in Bornovalova et al., 2020). The reliability score of DP relative to those of the subscale factors was similar to findings from previous work on general psychopathology factors in a bifactor model (see review by Scopel Hoffmann et al., 2022). Previous work suggests that children from low SES and ethnoracial minority backgrounds face elevated risks for childhood DP (Althoff et al., 2010; McQuillan et al., 2018), and the same demographic risk factors further align with elevated risk for mental health disorders and addictions at later ages (Evans & Cassells, 2014; Santiago et al., 2011). The present work builds upon these findings, suggesting that reductions in childhood DP may underlie how interventions for at-risk families may help to improve children’s later psychopathological risks and strengths-based outcomes.

Although the focus of this paper was on the DP and its correlates, a discussion of the AAA subscale factors is warranted. We note that the three subscale factors had satisfactory reliability as indicated by *ω* and the *H* coefficient. For the Anxiety/Depression and Attention Problem subscales, we may interpret that the two subscales are unique and differentiate from the DP, with at least half of item loadings above .4 and most above .3 for each factor. This would suggest that DP remains a general regulation-related difficulty, separate from the Anxiety/Depression and Attention Problems residuals that are dimensionally specific to the psychopathological symptom groups they each represent, for example, internalizing and attentional difficulties, respectively. However, we note that the Aggression subscale showed marginal reliability according to the *H* coefficient in this sample, while item-level factor loadings were often lower than expected and sometimes inconsistent in directionality (similar to findings in Haltigan et al., 2018). As noted by Bornovalova et al. (2020), this suggests an over-emphasis of Aggression items in the DP factor relative to the Aggression subscale factor, although the DP factor itself appears to be a balanced representation of the three difficulty domains. Additionally, as noted by Watts et al. (2019), the very low item-level loadings suggest a narrower characterization of the Aggression subscale. As the focus of the paper was on the DP rather than the AAA subscales, these lower loadings for the Aggression subscale were not deemed problematic. Yet, they should be given careful consideration in further research focusing on the unique components of AAA subscales.

Our second aim was to examine the potential parental antecedents and adolescent outcomes, particularly strengths-based outcomes, associated with heightened DP at childhood. With regards to antecedents, we found that mothers who were depressed had children with elevated DP while mothers using warmer parenting had children with reduced DP, echoing previous work with mostly White and well-educated families (e.g., Deutz et al., 2020). These links were further not significant for the majority of the AAA residuals, highlighting the effects of maternal depression and warmth on DP in particular. Contrary to expectations and previous work (e.g., Kim et al., 2012), maternal hostility was not significantly related to children’s DP in the present sample. However, there were few observed hostile parenting behaviors in general, suggesting a potential floor effect.

The relatively high warmth and low hostility demonstrated in the present sample generate several potential explanations. Although at-risk and fragile families have been historically pathologized as facing greater risk for maladaptive parenting and negative family functioning, it is possible that in reality many of these families are well-adjusted to parenting-related pressures. On the other hand, the null results in the present study may also be due to parents’ lesser use of yelling or physical discipline (spanking) with children in the presence of the research team, in an observed assessment. This effect may be explained by the stigma experienced by some low-income single mothers, such as about their SES and fears about their children being referred to Child Protective Services (Broussard et al., 2012), which may lead to efforts to demonstrate socially-acceptable forms of “good” parenting in the presence of researchers (Banister et al., 2016). However, some researchers consider opposite reasoning, namely, that low SES parents who are particularly distressed would be less skilled at “faking good” in their parenting or that they may underreport positive parenting due to low parenting confidence (Herbers et al., 2017). Alternatively, since low-income single mothers often continue to live with their parents (Banister et al., 2016), they may benefit from sufficient social support and help with childcare, aiding in their habitual use of more positive parenting characteristics that benefit child adjustment, such as less hostile parenting (DeLeire & Kalil, 2002).

Our results on parenting effects may also be due to the diversity in ethnoracial groups captured in the present sample. For example, Weis and Toolis (2010) found that low-SES African American mothers and high-SES Latina mothers were the most hostile towards their children, while European American mothers of any SES were least likely to be hostile (while simultaneously most likely to be warm). Potentially, the presence of warmth and relative absence of hostility in our results is due to the offsetting of higher or lower levels of each type of parenting across the ethnic subgroups in our sample. However, previous research has also found contrary effects particularly with regards to links between hostility and child outcomes, with parent physical punishment-based hostility related to children’s behavioral dysregulation (in the form of misconduct) to an equal degree across European and African Americans (Wang & Kenny, 2014). Regardless, given the biases present in both observed and self-reported hostile parenting measures, a complementary approach to measuring negative parenting behavior may be through child-reported parenting, which tends to show effects with child outcomes beyond other informant measures (Robichaud et al., 2020).

With regards to the unique variance captured by the AAA residuals rather than the DP factor, mothers who were depressed and used less hostile parenting had children with elevated anxiety/ depression residuals. These findings are in line with previous work by Deutz et al. (2020), which found that maternal depression significantly predicted less harsh controlling parenting and trended (albeit nonsignificantly) towards predicting heightened anxiety/depression residuals. These results may be a consequence of depressed mothers’ biased cognitions about their child: compared to nondepressed mothers, mothers with clinical depression have been found to report more emotional problems in their children (Weissman et al., 2004). Further, a gene × environment explanation for these findings would highlight that children may possess a genetic risk for internalizing difficulties, perhaps inherited from clinically depressed mothers, and when mothers’ parenting is affected by depressive symptoms (e.g., child maltreatment) children can face higher risk for experiencing anxiety and depression related problems as they develop (e.g., Cicchetti et al., 2010; Hayden et al., 2010). There is also evidence that DP itself may be heritable (see Doyle et al., 2010).

However, in light of the bifactor structure found in our study, an alternative explanation is possible. Within a bifactor structure, the DP represents a cross-symptomatic index of regulation difficulties, while the AAA residuals may represent amplitude or intensity of arousal present in each symptom group. For example, the anxiety/depression residual may represent intensity of emotional arousal in children, such as intense crying or visibly apparent tenseness. In this light, the present findings demonstrate that depressed or less overtly hostile mothers – who may be less emotionally responsive – have children that show more intense expressions of anxiety and depression. These findings are in line with previous research showing that when mothers are less emotionally expressive (e.g., due to depression), children show heightened emotion expression such as more apparent or frequent crying in order to elicit their mothers’ attention or to initiate self-directed regulation (see Goodman & Gotlib, 1999). Nevertheless, further work is needed to understand the meaningfulness of the AAA residuals in the context of the bifactor model, after accounting for the unique components captured by the DP. In particular, an examination of different outcomes that more specifically relate to the unique variance represented by the AAA residuals may be warranted.

Regarding adolescent outcomes, we found the expected associations between heightened childhood DP and more anxiety, depression, and impulsivity at adolescence, which confirmed previous work with children from White and moderate-high SES backgrounds (e.g., Deutz et al., 2020). Importantly, extending on existing literature and in line with our hypotheses, children with heightened DP also reported less adaptive social skills, perseverance, and optimism in adolescence. The same effects were not found with AAA residuals, suggesting that DP has unique associations to child outcomes independent from each individual AAA residuals. The findings help to broaden our existing understanding of the potential longitudinal outcomes of DP to encompass psychosocial strengths. Although this is one of the first works to examine strengths-based adolescent outcomes of DP, the results are in line with existing work on childhood self-regulation and related outcomes. For example, a recent meta-analysis reported that childhood self-regulation is related to concurrent and later social competence (Robson et al., 2020). Further, children who better self-regulate have been found to use more active coping strategies to deal with stressors (Lengua & Long, 2002), which may allow for psychosocial flourishing. Although no significant links were found between DP and engagement, connection or happiness, the directionality of these links were also largely in the expected direction. Thus, overall, childhood DP appears to be linked to reduced psychosocial strengths and enhanced internalizing difficulties and impulsivity at adolescence in at-risk fragile families.

Our findings suggest that DP is important across SES and ethnoracial backgrounds, especially given that it is also associated with strengths-based outcomes. Based on the results of this study, it may be possible to identify how specific parental or child factors could be targeted to improve strengths outcomes in children from these and other backgrounds. For example, for those families in which parental factors and child DP both appear to have independent influences on adolescent outcomes, addressing child-level (e.g., school-based) self-regulation training (Kurdi et al., 2022) alongside parent-level intervention (e.g., Gabalda et al., 2010) may be beneficial. One way to intervene may be through increasing social support for low-income single parents to alleviate their parenting and financial stresses: Choi and Pyun (2014) reported in a sample of single mothers using the same dataset that nonresident fathers’ financial and instrumental support reduces mothers’ parenting stress, improves their positive parenting, and facilitates children’s positive cognitive and behavioral outcomes. Combined, these findings suggest specific areas for targeted intervention that may benefit families with different needs. However, more work is needed to decipher and disentangle the multiplicity of effects that determine heightened DP in childhood to address specific targets for intervention.

A number of limitations must be noted in this study. First, psychosocial strengths measures were not available at age 9, and thus could not be controlled in the model testing longitudinal links between DP and adolescent strengths-based outcomes. A limited number of observed parenting variables were available at age 5, and future work including fathers’ parenting behavior is needed to shed some light on their unique role in the development of childhood DP. Although encompassing Black and Hispanic Americans as the majority of the sample, this study had a small proportion of other non-White ethnoracial groups. Further work is needed to determine the relevance of DP in other groups, such as immigrant and Asian American families. In particular, DP may manifest differently in children of Asian American backgrounds given that individuals from these groups have been shown to experience heightened somatization problems (Kim et al., 2019; Ryder et al., 2008). It is possible that, for these groups, somatic symptoms could be included in their DP assessments, and examining this possibility would be clinically important for serving diverse populations. Further, researchers have proposed that children’s internalizing symptoms may represent a form of overregulated compliance with adults, including parents (Eisenberg et al., 2001). Thus, comparing the DP with other configurations of regulation difficulties, such as overregulation and underregulation, in other diverse samples may be well worthwhile.

Regardless of these limitations, the present findings serve to broaden the relevance and clinical usability of the DP to children of at-risk fragile families marked by single parenthood, low SES, and ethnoracial minority backgrounds. Despite recently increasing attention on the DP as a precursor to future indices of psychopathology (Bellani et al., 2012), the majority of research on the DP has focused on White and European-origin families and maladjusted outcomes at adolescence and adulthood. The present work addresses these issues by shedding light on the parental risk factors and adolescent adjustment outcomes that may be associated with heightened DP in underrepresented families in clinical psychological research. Finally, these findings also suggest that childhood DP may predict future difficulties in the development of psychosocial strengths over the course of development.

## Supplementary Material

1

## Figures and Tables

**Table 1.** Descriptive statistics, interitem consistency, and correlations for variables of interest

	1	2	3	4	5	6	7	8	9	10	11	12	13	14	15	16
Parental factors (age 5)																
1. Warmth																
2. Hostility	−.28**															
3. Depression	−.04	.08**														
CBCL problem subscales (age 9)																
4. Anxious/depressed	−.01	.03	.16**													
5. Attention	−.08**	.09**	.13**	.51**												
6. Aggression	−.13**	.16**	.17**	.60**	.66**											
7. DP	−.10**	.12**	.18**	.78**	.85**	.92**										
Adolescent outcomes (age 15)																
8. Adaptive social skills	.13**	−.06*	− .05*	−.07**	−.11**	−.09**	−.11**									
9. Engagement	−.05*	.03	.00	.01	.05*	.05*	.04*	.14**								
10. Perseverance	.00	.01	−.03	−.09**	−.08**	−.07**	−.09**	.35**	.12**							
11. Optimism	.02	−.02	−.03	−.05*	−.06*	−.05*	−.06**	.36**	.15**	.50**						
12. Connection	.05	−.03	−.02	−.05*	−.06*	−.04	−.06*	.39**	.16**	.46**	.43**					
13. Happiness	.03	−.01	—.06*	−.07**	−.05*	−.05*	−.06**	.41**	.13**	.47**	.53**	.53**				
14. Anxiety	−.05	.00	.04	.10**	.11**	.10**	.12**	−.24**	.22**	−.25**	−.25**	−.20**	−.34**			
15. Depression	−.04	−.02	.04*	.08**	.09**	.09**	.10**	−.34**	.07**	**36	−.41**	−.40**	−.65**	.65**		
16. Impulsivity	−.12**	.07**	.05*	.08**	.14**	.18**	.17**	−.14**	.35**	−.17**	−.06**	−.07**	−.13**	.45**	.32**	
*M*	.77	.10	-	.18	.37	.25	.26	2.41	2.98	3.56	3.41	3.77	3.58	1.82	1.60	2.48
*SD*	.26	.21	-	.22	.36	.28	.24	.33	.62	.42	.50	.37	.50	.65	.60	.69
Min	0.0	0.0	0.0	0.0	0.0	0.0	0.0	1.0	1.0	1.5	1.0	1.0	1.0	1.0	1.0	1.0
Max	1.0	1.0	1.0	2.0	2.0	2.0	2.0	3.0	4.0	4.0	4.0	4.0	4.0	4.0	4.0	4.0
*α*	.79	.58	-	.78	.85	.89	.93	.75	.59	.65	.55	.63	.75	.76	.75	.78

*Note*. CBCL Problem Subscales represent calculated means not based on factor analysis.

* *p* < .05

** *p* < .01.

**Table 2.** Model fit statistics for the three tested factor structures of the DP

								Factor reliability							
				RMSEA CI				DP		AD		AT		AG	
	*χ* ^2^	df	RMSEA	Lower	Upper	CFI	WRMR	*ω*	*H*	*ω*	*H*	*ω*	*H*	*ω*	*H*

Bifactor	2588.692	738	.035	.034	.036	.950	1.636	.97	.97	.92	.76	.92	.75	.96	.69

Second order	4557.701	776	.049	.047	.050	.897	2.337								

One factor	5134.947	779	.052	.051	.054	.881	2.499								

*Note*. RMSEA CI Lower and Upper represent 90% confidence interval lower and upper bounds, respectively. Factor reliability ω represents omega, H represents the *H* coefficient. DP represents the Dysregulation Profile factor, AD represents the Anxiety/Depression factor, AT represents the Attention Problems factor, and AG represents the Aggression factor.

## Data Availability

Data pertaining to this study are available from the Fragile Families and Child Well-being Study website, fragilefamilies.princeton.edu.
