# Supplementary material for: Dysregulation profile in children of ethnoracially diverse at-risk families: Factor structure and longitudinal correlates
Source: Dev Psychopathol. Author manuscript; Available in PMC 2024 Nov 1. (PMC10464460; doi:10.1017/S095457942300007X)
Supplement: 1 [file NIHMS1866811-supplement-1.pdf]

Supplementary Table 1.

Factor loadings for the one-factor, second-order, and bifactor models

Items	One factor	Second Order			Bifactor			
		AD	AT	AG	DP	AD	AT	AG
AD								
Child is nervous, highstrung, or tense	**.64**	**.71**			**.60**	**.53**		
Child is self-conscious or easily embarrassed	**.66**	**.74**			**.51**	.35		
Child is too fearful or anxious	**.53**	**.60**			**.57**	**.63**		
Child cries a lot	**.66**	**.73**			**.59**	.03		
Child fears certain animals/situations/places other than school	**.57**	**.63**			.38	.29		
Child fears going to school	**.41**	**.46**			**.65**	.36		
Child fears he or she might do something bad	**.68**	**.75**			**.49**	**.46**		
Child feels he or she has to be perfect	**.54**	**.61**			.21	**.48**		
Child feels or complains that no one loves him or her	.27	.31			**.63**	.33		
Child feels worthless or inferior	**.65**	**.72**			**.67**	**.41**		
Child feels too guilty	**.71**	**.78**			**.56**	**.51**		
Child talks about killing self	**.62**	**.69**			**.73**	.26		
Child worries	**.74**	**.81**			**.44**	**.51**		
AT								
Child has nervous movements or twitches	**.83**		**.90**		**.46**		.34	
Child fails to finish things he or she starts	**.50**		**.53**		**.56**		.38	
Child can't concentrate, can't pay attention for long	**.51**		**.55**		**.57**		**.73**	
Child can't sit still, is restless, or hyperactive	**.62**		**.67**		**.59**		**.43**	
Child is confused or seems to be in a fog	**.73**		**.78**		**.56**		**.48**	
Child day-dreams or gets lost in thoughts	**.66**		**.71**		**.51**		**.47**	
Child is impulsive or acts without thinking	**.64**		**.69**		**.74**		.28	
Child does poor school work	**.59**		**.63**		**.55**		.39	
Child is inattentive or easily distracted	**.76**		**.81**		**.67**		**.54**	
Child stares blankly	**.61**		**.65**		**.62**		.34	
AG								
Child smokes, chews, or sniffs tobacco	**.77**			**.79**	**.63**			.13

Child is cruel, bullies, or shows meanness to others	**.66**	**.68**	**.70**	.35
Child demands a lot of attention	**.62**	**.63**	**.67**	.04
Child destroys his or her own things	**.73**	**.75**	**.75**	**.40**
Child destroys things belonging to family or others	**.65**	**.66**	**.72**	**.47**
Child is disobedient at home	**.79**	**.81**	**.63**	**.48**
Child is disobedient at school	**.78**	**.80**	**.57**	**.58**
Child gets in many fights	**.67**	**.69**	**.65**	**.43**
Child physically attacks people	**.64**	**.66**	**.72**	.33
Child screams a lot	**.70**	**.72**	**.73**	.09
Child is stubborn, sullen, or irritable	**.75**	**.77**	**.76**	.01
Child has sudden changes in mood or feelings	**.71**	**.73**	**.78**	-.11
Child sulks a lot	**.73**	**.74**	**.75**	-.30
Child is suspicious	**.73**	**.75**	**.69**	-.23
Child teases a lot	**.68**	**.70**	**.65**	.21
Child has temper tantrums or a hot temper	**.63**	**.65**	**.75**	.21
Child threatens people	**.65**	**.67**	**.80**	.37
Child is unusually loud	**.75**	**.76**	**.68**	.02

Supplementary Table 2.

Path estimates for full model (Unstandardized estimates)

IV	DV	B	SE	p
Warmth (T1)	DP (T2)	-.149	.070	.034
Hostility (T1)	DP (T2)	.119	.085	.160
Depression (T1)	DP (T2)	.164	.041	.000
Warmth (T1)	ANX (T2)	.163	.084	.053
Hostility (T1)	ANX (T2)	-.226	.110	.039
Depression (T1)	ANX (T2)	.106	.047	.023
Warmth (T1)	ATT (T2)	.013	.049	.799
Hostility (T1)	ATT (T2)	-.045	.058	.436
Depression (T1)	ATT (T2)	-.008	.030	.794
Warmth (T1)	AGG (T2)	-.024	.018	.177
Hostility (T1)	AGG (T2)	.040	.025	.107
Depression (T1)	AGG (T2)	-.003	.006	.638
DP (T2)	Social Skills (T3)	-.040	.016	.010
ANX (T2)	Social Skills (T3)	.014	.025	.580
ATT (T2)	Social Skills (T3)	-.056	.031	.076
AGG (T2)	Social Skills (T3)	.403	.252	.110
Warmth (T1)	Social Skills (T3)	.136	.035	.000
Hostility (T1)	Social Skills (T3)	-.025	.045	.570
Depression (T1)	Social Skills (T3)	-.018	.019	.332
DP (T2)	Engagement (T3)	.047	.029	.111
ANX (T2)	Engagement (T3)	-.038	.047	.423
ATT (T2)	Engagement (T3)	.034	.060	.571
AGG (T2)	Engagement (T3)	-.010	.289	.971
Warmth (T1)	Engagement (T3)	-.087	.068	.201
Hostility (T1)	Engagement (T3)	.038	.077	.620
Depression (T1)	Engagement (T3)	-.005	.039	.898
DP (T2)	Perseverance (T3)	-.052	.020	.008
ANX (T2)	Perseverance (T3)	-.021	.032	.501
ATT (T2)	Perseverance (T3)	.006	.039	.874
AGG (T2)	Perseverance (T3)	.365	.257	.157
Warmth (T1)	Perseverance (T3)	.049	.042	.245
Hostility (T1)	Perseverance (T3)	.000	.051	.997
Depression (T1)	Perseverance (T3)	-.003	.026	.893
DP (T2)	Optimism (T3)	-.052	.023	.024
ANX (T2)	Optimism (T3)	.039	.036	.281

ATT (T2)	Optimism (T3)	.013	.047	.774
AGG (T2)	Optimism (T3)	.319	.284	.261
Warmth (T1)	Optimism (T3)	.056	.052	.278
Hostility (T1)	Optimism (T3)	-.040	.063	.524
Depression (T1)	Optimism (T3)	-.014	.030	.652
DP (T2)	Connectedness (T3)	-.004	.018	.838
ANX (T2)	Connectedness (T3)	-.022	.028	.433
ATT (T2)	Connectedness (T3)	-.023	.033	.480
AGG (T2)	Connectedness (T3)	.145	.171	.398
Warmth (T1)	Connectedness (T3)	.066	.038	.083
Hostility (T1)	Connectedness (T3)	-.033	.050	.509
Depression (T1)	Connectedness (T3)	.009	.023	.687
DP (T2)	Happiness (T3)	-.039	.023	.085
ANX (T2)	Happiness (T3)	-.009	.036	.808
ATT (T2)	Happiness (T3)	-.014	.045	.762
AGG (T2)	Happiness (T3)	.504	.346	.144
Warmth (T1)	Happiness (T3)	.076	.052	.145
Hostility (T1)	Happiness (T3)	-.031	.064	.627
Depression (T1)	Happiness (T3)	-.039	.029	.175
DP (T2)	Anxiety (BSI) (T3)	.081	.030	.007
ANX (T2)	Anxiety (BSI) (T3)	.016	.050	.746
ATT (T2)	Anxiety (BSI) (T3)	.072	.066	.276
AGG (T2)	Anxiety (BSI) (T3)	-.252	.338	.457
Warmth (T1)	Anxiety (BSI) (T3)	-.136	.067	.044
Hostility (T1)	Anxiety (BSI) (T3)	-.062	.087	.482
Depression (T1)	Anxiety (BSI) (T3)	.016	.040	.691
DP (T2)	Depression (CESD) (T3)	.084	.027	.002
ANX (T2)	Depression (CESD) (T3)	-.042	.045	.351
ATT (T2)	Depression (CESD) (T3)	.035	.059	.557
AGG (T2)	Depression (CESD) (T3)	-.738	.469	.116
Warmth (T1)	Depression (CESD) (T3)	-.094	.062	.131
Hostility (T1)	Depression (CESD) (T3)	-.099	.084	.240
Depression (T1)	Depression (CESD) (T3)	.026	.035	.467
DP (T2)	Impulsivity (T3)	.138	.033	.000
ANX (T2)	Impulsivity (T3)	-.072	.051	.160
ATT (T2)	Impulsivity (T3)	.025	.067	.704
AGG (T2)	Impulsivity (T3)	.503	.406	.215
Warmth (T1)	Impulsivity (T3)	-.208	.073	.004
Hostility (T1)	Impulsivity (T3)	-.004	.087	.968
Depression (T1)	Impulsivity (T3)	.025	.041	.547
